# Functional Disassociation Between the Protein Domains of MSMEG_4305 of *Mycolicibacterium smegmatis* (*Mycobacterium smegmatis*) *in vivo*

**DOI:** 10.3389/fmicb.2020.02008

**Published:** 2020-08-19

**Authors:** Bożena Czubat, Alina Minias, Anna Brzostek, Anna Żaczek, Katarzyna Struś, Jolanta Zakrzewska-Czerwińska, Jarosław Dziadek

**Affiliations:** ^1^Department of Experimental and Clinical Pharmacology, University of Rzeszów, Rzeszów, Poland; ^2^Laboratory of Genetics and Physiology of Mycobacterium, Institute of Medical Biology, Polish Academy of Sciences, łLódź, Poland; ^3^Institute of Medical Sciences, Medical College of Rzeszów University, Rzeszów, Poland; ^4^Department of Bioenergetics, Food Analysis and Microbiology, Institute of Food Technology and Nutrition, University of Rzeszów, Rzeszów, Poland; ^5^Department of Molecular Microbiology, University of Wrocław, Wrocław, Poland

**Keywords:** *Actinomycetales*, protein domains, *Mycolicibacterium*, *Mycobacterium*, *smegmatis*, MSMEG_4305, vitamin B12

## Abstract

MSMEG_4305 is a two-domain protein of *Mycolicibacterium smegmatis* (*Mycobacterium smegmatis*) (*Mycolicibacterium smegmatis*). The N-terminal domain of MSMEG_4305 encodes an RNase H type I. The C-terminal domain is a presumed CobC, predicted to be involved in the aerobic synthesis of vitamin B12. Both domains reach their maximum at distinct pH, approximately 8.5 and 4.5, respectively. The presence of the CobC domain influenced RNase activity *in vitro* in homolog Rv2228c. Here, we analyzed the role of MSMEG_4305 in vitamin B12 synthesis and the functional association between both domains *in vivo* in *M. smegmatis*. We used knock-out mutant of *M. smegmatis*, deficient in MSMEG_4305. Whole-cell lysates of the mutants strain contained a lower concentration of vitamin B12, as it determined with immunoenzimatic assay. We observed growth deficits, related to vitamin B12 production, on media containing sulfamethazine and propionate. Removal of the CobC domain of MSMEG_4305 in Δ*rnhA* background hardly affected the growth rate of *M. smegmatis in vivo*. The strain carrying truncation showed no fitness deficit in the competitive assay and it did not show increased level of RNA/DNA hybrids in its genome. We show that homologs of MSMEG_4305 are present only in the *Actinomycetales* phylogenetic branch (according to the old classification system). The domains of MSMEG_4305 homologs accumulate mutations at a different rate, while the linker region is highly variable. We conclude that MSMEG_4305 is a multidomain protein that most probably was fixed in the phylogenetic tree of life due to genetic drift.

## Introduction

*Mycolicibacterium smegmatis* is a saprophytic bacterium found mainly in soil, water, and seborrheic secretions. It is widely regarded as a non-pathogenic organism, although in rare cases, it can cause skin and soft tissue infections (Reyrat and Kahn, 2001). *M. smegmatis* is relatively fast-growing. It is often used as a surrogate model to investigate the biology of slowly growing *Mycobacterium tuberculosis*. Recently the genus of *Mycobacterium* was amended new taxonomic order. The results of the phylogenetic analyses based on amino acid similarity support the existence of five novel clades: *Mycobacterium*, *Mycolicibacterium*, *Mycolicibacter*, *Mycolicibacillus*, and *Mycobacteroides* (Gupta et al., 2018).

**FIGURE 1:**
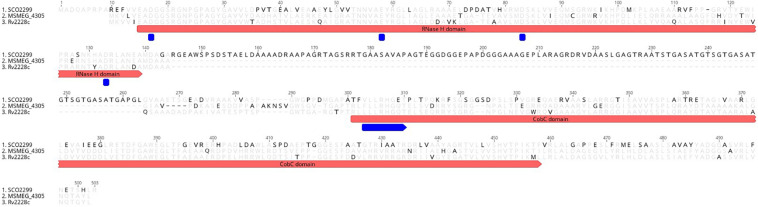
The alignment between protein sequences of MSMEG_4305 of *M. smegmatis* mc^2^, Rv2228c of *M. tuberculosis* H37Rv and SCO2299 of *S. coelicolor*. The range of the domains is marked with red. The active site residues are marked with blue. The disagreements between the alignments are highlighted in black.

One of the proteins of *M. smegmatis* is a two-domain protein MSMEG_4305. The N-terminal domain encodes an RNase H type I. The C′ terminal domain is a CobC domain, and it contains a signature motif of phosphoglycerate mutase family, [LIVM]-x-R-H-G-[EQ]-x-{Y}-x-N, as predicted by Prosite (Figure 1). First reports of this type of protein regarded the identification of RNase HI in *Corynebacterium glutamicum*, where the product of the gene rescued a temperature-sensitive phenotype of the RNase H mutant (Hirasawa et al., 2003). Subsequent structural studies with homologs SCO2299 from *Streptomyces coelicolor* (Ohtani et al., 2005) and Rv2228c from *M. tuberculosis* (Watkins and Baker, 2010) showed that the RNase H domain shares the archetypal RNase H fold conserved across the entire family of RNases H. The authors found that the protein has several specific characteristics. Unlike other RNases H, Rv2228c/N forms a dimer in the crystal structure. Rv2228c/N lacks basic protrusion in its structure, which in other RNases contacts the RNA/DNA substrate. Rv2228c/N also lacks a phosphate-binding pocket, believed to confer specificity for DNA, by requiring a nucleotide conformation that is accessible only to DNA.

Activity tests were performed on recombinant SCO2299 (Ohtani et al., 2005), Rv2228c (Watkins and Baker, 2010) and on MSMEG_4305 (Jacewicz and Shuman, 2015). The authors found that the N-terminal domains of all of these enzymes show RNase H activity, with minor differences in their specificity. SCO2299 enzyme cleaved RNA/DNA substrates and RNA–DNA junction (a junction between the 3′ side of RNA and 5′ side of DNA) of an Okazaki fragment-like substrate. Rv2228c displayed activity for RNA/DNA and RNA/RNA duplexes. MSMEG_4305 showed RNase H activity only for RNA/DNA duplexes, with no activity for RNA/RNA, DNA/DNA, nor single RNA strands. RNase H domain of SCO2299 and MSMEG_4305 reached maximum activity at approximate pH 8.5.

C-terminal domains of SCO2299, Rv2228c, and MSMEG_4305 showed phosphatase activity at acidic pH of approximately 4.5, and the activity nearly completely faded at pH ≥ 7. Hence, the authors determined the enzyme to be an acid phosphatase. For Rv2228c, the authors also tested the protein for α-ribazole-5-phosphatase, CobC, activity. The activity was detected using the Malachite green method of inorganic phosphate detection, and it confirmed that the enzyme does indeed have CobC activity.

The divergence in the activity level of N-terminal and C-terminal domains at distinct pH raises a question regarding the functional association between the domains within a single protein. N-terminal 140-aa domain of Rv2228c displayed significantly slower RNase activity compared to the full-length protein. Therefore, the authors suspected that the CobC domain might provide the function of the basic protrusion. Further, the authors suggested that the CobC domain may play a role in changing the conformation of the active site and thus increasing the efficiency of product release. They noted that their results stay in contrast to homologous SCO2299 from *S. coelicolor*, where the activities of the two domains, RNase H and acid phosphatase, are independent. They explained that these differences might be related to the length of the linker sequence between the domains. The interdomain linker in SCO2299 is approximately 120 residues, compared to 10 residues in Rv2228c. The short linker of Rv2228c might enable its C-terminal domain to modulate the activity of the N-terminal domain.

**FIGURE 2:**
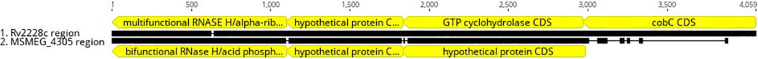
The alignment between genetic regions surrounding MSMEG_4305 and Rv2228c, in *M. smegmatis* and *M. tuberculosis*, respectively. The alternative CobC encoding gene is missing in the genome of *M. smegmatis*.

The RNase HI of MSMEG_4305 is not the sole RNase HI encoded by the genome of *M. smegmatis*. The bacterium also carries the *rnhA* gene (Minias et al., 2015a; Gupta et al., 2017). Each of the genes which encode RNases HI can be removed from the genome of *M. smegmatis*, but double deletion is not possible (Minias et al., 2015a; Gupta et al., 2017). In *M. tuberculosis*, functional annotation identifies only one gene encoding an RNase HI, Rv2228c. Mathematical models evaluating forces of natural selection indicated an important role of Rv2228c for the proper functioning of the cell. Rv2228c is influenced by purifying selective pressure. All of the codons under selection localize in the CobC domain of the protein (Minias et al., 2018). In early studies, it was not possible to obtain transposon mutants deficient in the product of this gene (Sassetti et al., 2003). Lamichhane et al. (2003) obtained the transposon mutant of Rv2228c, but the place of the insertion of the transposon was at the position 1088 of 1095 bp long gene. Finally, studies of DeJesus et al. (2017) showed that insertion in 17 sites of the gene causes growth deficit, while insertion in the remaining three sites is not significant. Hence the gene seems to be non-essential yet important for the bacterium. *M. tuberculosis* genome contains a second gene encoding predicted CobC, Rv2231c. Rv2231c is in the operon with Rv2228c (Caspi et al., 2012). The alignment of the gene sequences surrounding the Rv2228c and MSMEG_4305 show that potential additional CobC encoding gene is missing in *M. smegmatis* (Figure 2). As to the other two genes in the operon, Rv2230c encodes a predicted GTP cyclohydrolase, while Rv2229c encodes a protein of unknown function.

Here, we analyzed the role of MSMEG_4305 in vitamin B12 synthesis and the functional association between both domains of the protein *in vivo* in *M. smegmatis*. We show that the CobC domain of MSMEG_4305 is involved in vitamin B12 biosynthesis. Its absence does not significantly affect the functioning of the RNase H domain *in vivo*. Our results provide an experimentally confirmed example of a multidomain protein probably fixed in the phylogenetic tree of life due to genetic drift.

## Materials and Methods

### Bacteria and Culture Conditions

We obtained mutant *M. smegmatis* through the method of gene replacement by homologous recombination, as described previously (Minias et al., 2015a). Briefly, cells of *M. smegmatis* mc^2^ were transformed with a p2NIL derivative plasmid carrying the gene of interest with a large internal deletion. The plasmid recombined with the native gene, which resulted in the generation of mutants carrying a defective copy of the gene. The mutants were selected in the multistep selection process, and their genotype was confirmed by Southern blot. The mutants were further complemented with additional copies of the genes under inducible acetamidase promoter that were introduced at the *attB* site of the mycobacterial genome on derivatives of plasmid pMV306. Complementation of mutants was confirmed by Southern blot or by PCR The list of primers used in this study is presented in Supplementary Table S1. All molecular cloning was performed in *E. coli*. Cultures of *E. coli* T10 were carried out at 37°C for 18–20 h in liquid or solid Luria-Bertani broth. When necessary, the media were supplemented with antibiotics or other supplements at the following concentrations: kanamycin 50 μg/ml (BioShop); ampicillin 100 μg/ml (BioShop) and X-gal 40 μg/ml (BioShop).

Cultures of *M. smegmatis* were carried out in nutrient broth (NB) (Difco) or 7H9 broth (Becton, Dickinson, and Company) or minimal broth (Griffin et al., 2012) with or without oleic albumin dextrose catalase growth supplement (OADC) (Becton- Dickinson) and 0.05% Tween 80 (Sigma) at 37°C. When necessary, media were supplemented with antibiotics or other supplements at the following concentrations: cobalt chloride 12 μg/ml (Sigma Aldrich), kanamycin 25 μg/ml (BioShop); X-gal 40 μg/ml (BioShop), sucrose 2% (Sigma Aldrich), vitamin B12 10 μg/ml (Sigma Aldrich), propionate 0.1% (Sigma Aldrich), glucose 0.1% (BioShop), sulfamethazine sodium salt 600 μg/ml (Sigma Aldrich), tyloxapol 0.015% (Sigma-Aldrich). Tyloxapol is a detergent that cannot be utilized by mycobacteria as a carbon source (Zinniel et al., 2012). All cultures were started at OD_600_ = 0.05. To adjust the pH of growth media, we used HCl (Sigma Aldrich) or 1M NaOH (Sigma Aldrich), for acidic medium (pH = 5.5) or alkaline medium (pH = 10), respectively. Starved cultures used for experiments of detecting vitamin B12 in cell lysates were carried out in 7H9 broth supplemented with Tween 80 and CoCl_2_.

### Growth Curves

The growth curves of *M. smegmatis* strains were measured by estimating the number of colony-forming units (CFU) at specific time points by flow cytometry and by plating diluted batches of cultures on solid media. We used Guava EasyCyte Flow Cytometer (Merck) for flow cytometry. Unstained samples of bacterial cultures were serially diluted in freshly filtered 7H9 growth medium to reach optimal concentration between 400 to 800 cells per μl. The filtered 7H9 medium was used as a negative control to discriminate bacteria from debris. Each growth curve was drawn based on data from three independent experiments.

### Evaluation of Cell Length

Aliquots of cells were harvested at 24 h of culture in 7H9 broth supplemented with Tween 80, OADC, cobalt, and where applicable, with vitamin B12. The supernatant was removed by centrifugation, and the cell pellet was suspended in 1% paraformaldehyde (Sigma-Aldrich). Following centrifugation, cells were applied to microscope slides and observed using a light microscope Nikon Eclipse Ti. The image obtained from the microscope was analyzed using NIS Elements Analysis D 3.2 64-bit. For each strain, we analyzed data from three independent cultures. Alternatively, samples of cell cultures were analyzed on the flow cytometer Guava EasyCyte Flow Cytometer (Merck). Changes in the value of forward site scatter (FSC) are linked to changes in the size of the cells (Tzur et al., 2011).

### Detection of Vitamin B12 in Cell Lysates by ELISA

Samples of bacterial cultures were spinned down and resuspended in TE buffer [10 mM Tris–HCl pH = 8.0 (Serva) and 1 mM EDTA pH = 8.0 (Serva)] with the addition of 50 μL zirconia beads (BioSpec Products). Bacterial cells were broken twice using a Mini Beadbeater (MP Biomedicals) homogenizer. The cell sample was centrifuged again (14,000 rpm, 5 min, 4°C), and the obtained supernatant was transferred to a new Eppendorf tube. The cell lysates were stored at −80°C.

Total protein concentration in cell lysates was determined by the Bradford method (Bradford, 1976) using a DS-11 FX spectrophotometer (DeNovix) at a wavelength of 595 nm. Calculation of the protein concentration was based on a standard curve prepared using a solution of bovine albumin (Sigma-Aldrich) at a starting concentration of 10 mg/ml. This solution was diluted in a series of dilutions to give albumin concentrations of 5, 2.5, 1.25, 0.625, 0.312, and 0.156 mg/ml. Samples were used in ELISA assay if they contained at least 1 mg of protein/ml.

Measurement of vitamin B12 concentration in cell lysates was carried out using the commercial Vitamin B12 ELISA DEB12E01 (Demeditec Diagnostics GmbH). The assay used the standard vitamin B12 concentration (0; 0.4; 1.0; 4.0; 10.0; 40.0 ng/ml) that allowed to determine the standard curve and calculate the concentration of vitamin B12 in cell lysates. Briefly, 50 μl of cell lysates were applied to the wells of a 96-well plate. Next, 50 μl of vitamin B12 peroxidase conjugate was added. Plates were shaken for 60 min at room temperature. Then, the wells were washed three times with Washing Solution. 100 μl of the substrate was added to each well, and the plate was incubated for 20 min in the dark. During incubation, the substrate solution turned blue in the samples. After this time, the reaction was stopped by adding 100 μl Stop Solution. Within 30 min of completing the test, absorbance was measured at 450 nm using a Benchmark Plus 96-well plate spectrophotometer (BIO-RAD). All analyzes were carried out in three independent biological replicates and two technical replicates. The results were expressed in ng of vitamin B12 per mg of protein.

### Relative Fitness Evaluation

The analysis was performed as described previously (Gagneux et al., 2006). Fresh, logarithmic phase cultures were diluted to OD = 0.05 and mixed in equal proportions into one culture. At the time of the initiation of the experiment, control samples were plated on selective media containing either hygromycin or kanamycin. After 24 and 72 h of culture, diluted samples were plated on selective media, and the number of CFU was evaluated. The relative fitness, W, was calculated from the change in the relative abundances of the cells between the initial and final samples in the competition assay: *W* = ln(N Km 24 or 72/N Km 0)/ln (N Hyg 24 or 72/N Hyg 0), where N Km indicates population density grown on kanamycin plates, hence Δ*rnhA*Δ4305:*attB* + N’ter4305 mutant, 0 24 or 72 indicates an hour of culture, and N Hyg indicates population density grown on hygromycin plates, hence Δ*rnhA*Δ4305:*attB* + 4305 mutant strain. The results are mean values of relative fitness with standard deviations.

### Immunodetection of RNA/DNA Hybrids

The detection was carried out, as described previously (Minias et al., 2015b). Bacterial cultures were spun down after 24 h of growth and resuspended in TE buffer.[10 mM Tris–HCl (Serva), pH 8.0 and 1 mM EDTA (Serva)]. Zirconia beads (BioSpec Products) were added and the samples were homogenized using an MP Fast Prep homogenizer. Subsequently, an equal volume of phenol: chloroform (Sigma) was added, and after vigorous shaking, the samples were centrifuged at 14000 × *g* for 10 min. The aqueous phase was transferred to a new Eppendorf tube, and three volumes of ethanol (Sigma) were added. The samples were incubated at −20°C overnight and centrifuged for 10 min at 14000 × *g*. The pellet was dried at room temperature for approximately 15 min and resuspended in water. The samples were serially diluted, and 2 μl of each dilution was placed onto an Amersham Hybond N + nylon membrane (GE Healthcare Life Sciences). The membrane was air-dried and blocked with 5% milk in PBST [137 mM NaCl (Sigma); 2.7 mM KCl (Sigma); 10 mM Na2HPO4 2 H2O (Sigma); 2 M KH2PO4 (Sigma) pH 7.4; and 0.02% Tween 20 (Sigma)] at room temperature. The membrane was washed three times in PBST for 5 min at room temperature and incubated overnight at 4°C with a primary monoclonal mouse antibody against the RNA/DNA hybrid (D5H6) (Covalab) suspended in PBST containing 1% bovine serum albumin. Subsequently, the membrane was washed three times in PBST at room temperature and incubated with a secondary anti-mouse rabbit IgG antibody (Sigma) conjugated with peroxidase suspended in PBST containing 1% milk at room temperature. The membrane was washed three times in PBST and covered with ECL detection reagent (Amersham Biosciences). The excess reagent was removed, and the membrane was tightly wrapped in Saran wrap and exposed to X-ray film (Thermo Scientific) for 1 min. The film was developed using a Kodak Medical X-ray Processor.

### Immunoprecipitation of RNA/DNA Hybrids

We performed immunoprecipitation of RNA/DNA hybrids according to a protocol described previously (García-Rubio et al., 2018) with minor modifications. We disrupted the cells by bead-beating three times for 20 s at a rate of 6/ms. DNA spooled on Pasteur pipette was dissolved in TE buffer and digested overnight with *Ava*I. The average size of fragments generated after cutting reference genome of NC_008596 *M. smegmatis* mc^2^ by *Ava*I is 505 bp. The amount of fragments containing RNA/DNA hybrids was estimated by qPCR in three genes belonging to distinct functional groups- *radA* involved in DNA metabolism, *cobL* involved in vitamin B12 biosynthesis, and *accD4* involved in synthesis of mycobacterial cell wall (Supplementary Table S1). qPCR was performed using SYBR Green PCR Master Mix (Thermo Fisher Scientific) on 7900HT real time PCR system (Applied Biosystems). Real-time PCR conditions were as follows: UDG activation 50°C for 2 min, initial activation at 95°C for 10 min, followed by 40 cycles at 95°C for 15 s (denaturation), 63°C for 30 s (annealing), 72°C for 30 s (extension). The enrichment of the precipitate was normalized in reference to corresponding samples before immunoprecipitation. Each immunoprecipitation was performed in three biological replicates. Each qPCR sample was analyzed in technical triplicate.

### Variability of MSMEG_4305 Homologs

The presence of homologs of MSMEG_4305 in genomes across the phylogenetic tree of life was determined using an online web resource STRING (Szklarczyk et al., 2017). The sequences of homologs of MSMEG were extracted from the genomes of bacteria obtained from the NCBI Genome Database (Supplementary Table S2). The sequences were downloaded, extracted, and aligned with Geneious R11 (Biomatters, Auckland, New Zealand). The amino- acid range of protein domains was estimated with SMART protein domain annotation resource (Letunic and Bork, 2018). Amino acid gaps were removed from the alignment remaining amino acids were concatenated. Sequence variability was assessed with DnaSP v6 (Rozas et al., 2017). The overall mean distance between sequences was estimated using MEGA with the p-distance model, with 10000 bootstrapping (Tamura et al., 2013). The level of synonymous (dS) and non-synonymous (dN) nucleotide substitutions was estimated with Datamonkey Online Server (Delport et al., 2010) with SLAC method, which uses a combination of maximum-likelihood (ML) and counting approaches to infer non-synonymous (dN) and synonymous (dS) substitution rates on a per-site basis for a given coding alignment.

### Statistical Analysis

Unless described otherwise, all statistical analyses were performed with Statistica 13 (StatSoft, Tulsa, OK, United States). Statistical analysis was performed by comparing cell density at different time points by one-way ANOVA and Dunnett’s *post hoc* or Student’s *t*-test. The cut-off limit of statistical significance was *p* < 0.05.

## Results

### Homologs of MSMEG_4305 Across the Phylogenetic Tree of Life

**FIGURE 3:**
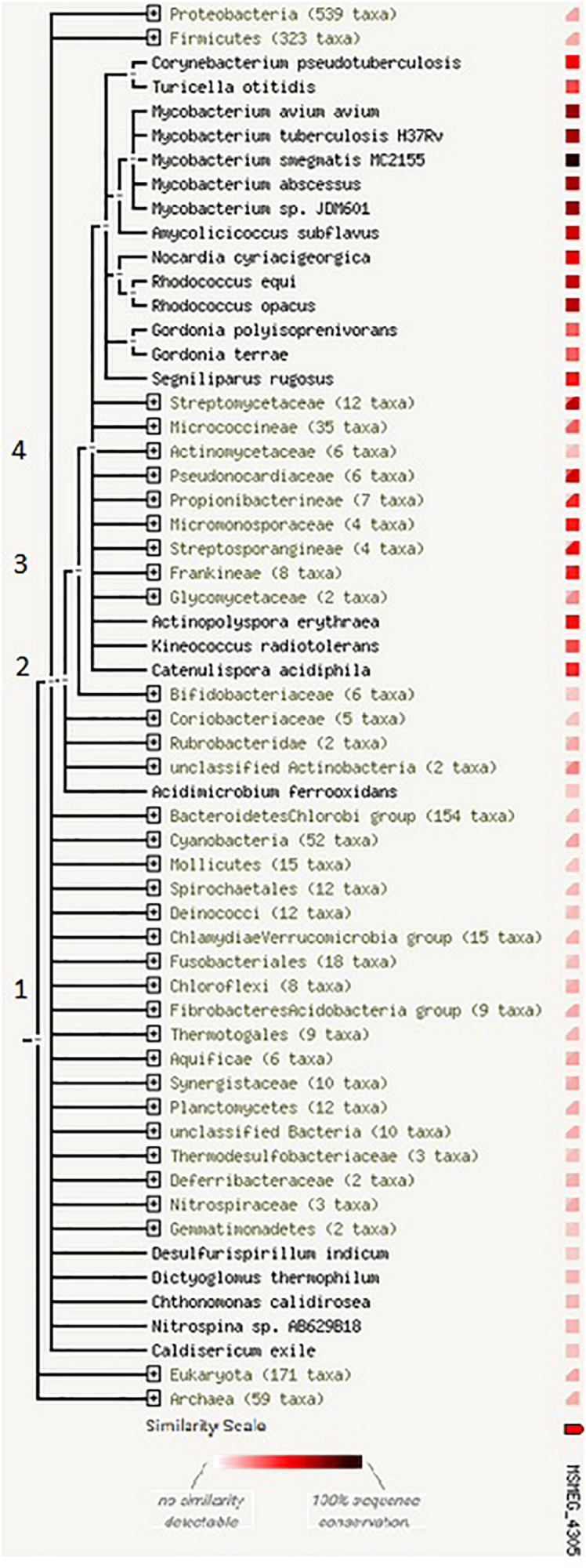
The occurrence of homologs of MSMEG_4305 across the phylogenetic tree. The tree is expanded at nodes (1) cellular organisms (2) bacteria (3) *Actinobacteria* (4) *Actinomycetales*. The legend beneath the phylogenetic tree shows colors that denote the similarity level of its best hit in a given STRING genome. For groups of genomes that are collapsed in the phylogenetic tree, two distinct colors indicate the lowest and the highest similarity observed within that clade.

The analysis by STRING protein showed that homologs of MSMEG_4305 are present only in bacteria in *Actinomycetales* phylogenetic branch [according to the old classification system (Gao and Gupta, 2012; Figure 3)]. This observation suggests that the genes homologous to MSMEG_4305 descended from the same ancestral sequence that arose in a phylogenetic tree relatively recently. The ortholog of MSMEG_4305 is not present in *Actinomycetaceae*, while it is present in both flanking branches of the tree.

To investigate the level of variability across orthologs of MSMEG_4305, we compiled a set of sequences derived from 87 species of *Actinomycetales* (Supplementary Figure S1). The shortest sequence was found in *Mycolicibacterium neoaurum* (1059 nucleotides), while *Nocardia terpenica* (1356 nucleotides) contained the longest sequence. There were 614 segregating sites in the sequences, with the total number of mutations of 1435. We observed 82 haplotypes among the sequences. Haplotype diversity was 0.996, while nucleotide diversity was 0.33. The average number of nucleotide differences was 244.3.

We analyzed the level of nucleotide substitutions across RNase H domains and CobC domains of homologs of MSMEG_4305. The mean level of dN-dS in CobC domains was −0.724, while the mean level of dN-dS in RNase H domains was −1.099. Further, the overall mean distance between the sequences of CobC domains was 0.449 ± 0.024 SE. The overall mean distance between RNase H domains was 0.316 ± 0.028 SE.

The linker region contained, on average, 55.5 ± 3.13 SE residues. We found *Streptomyces* sp. Z022 linker to be the longest, with 162 residues. The shortest linker of *Mycolicibacterium neoaurum* was 24 amino-acids long. Accordingly, the linker length of *M. smegmatis* mc^2^ is 37 residues, and *M. tuberculosis* H37Rv is 36 residues. Among 87 species of *Actinomycetales*, on average, the sequences showed 20.663% ± 0.142 SE amino acid similarity.

### Deletion of MSMEG_4305 Does Not Affect the Growth Rate, but It Does Affect the Size of the Cells

We previously obtained in our laboratory Δ4305 mutant (Minias et al., 2015a). As a control strain, we generated knock out mutants of the *cobIJ* gene (Supplementary Figure S2). The *cobIJ* gene catalyzes two methylation steps, of precorrin-2 C20 methyltransferase [CobI; EC:2.1.1.130] and precorrin-3B C17-methyltransferase [CobJ, EC:2.1.1.131] of the cobalamin biosynthetic pathway. It is essential for vitamin B12 synthesis (Raux et al., 2000). Guzzo et al. (2016) confirmed the role of *cobIJ* in vitamin B12 biosynthesis in *M. smegmatis*. Additional control strains were knock out mutants complemented with full copies of the deleted genes inserted at the *attB* site of the mycobacterial genome (Supplementary Figures S2, S3).

**FIGURE 4:**
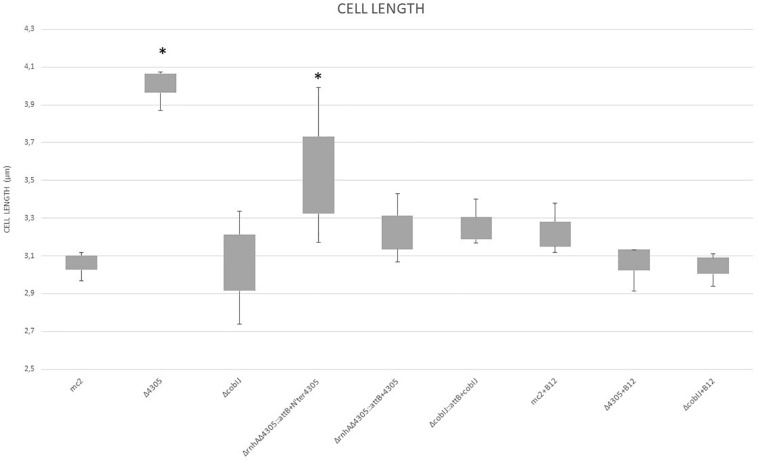
The comparison of cell length of *M. smegmatis* strains grown in 7H9 broth supplemented with cobalt chloride, OADC and Tween 80, with or without vitamin B12. The data are representative of three independent experiments. Statistical analysis was performed by comparison of the cell size of analyzed strains with *M. smegmatis* mc^2^ by one-way ANOVA and Dunnett’s *post hoc*. The cut-off level of statistical significance was *p* < 0.05. An asterisk marks values statistically different from the wild type *M. smegmatis* mc^2^.

**FIGURE 5:**
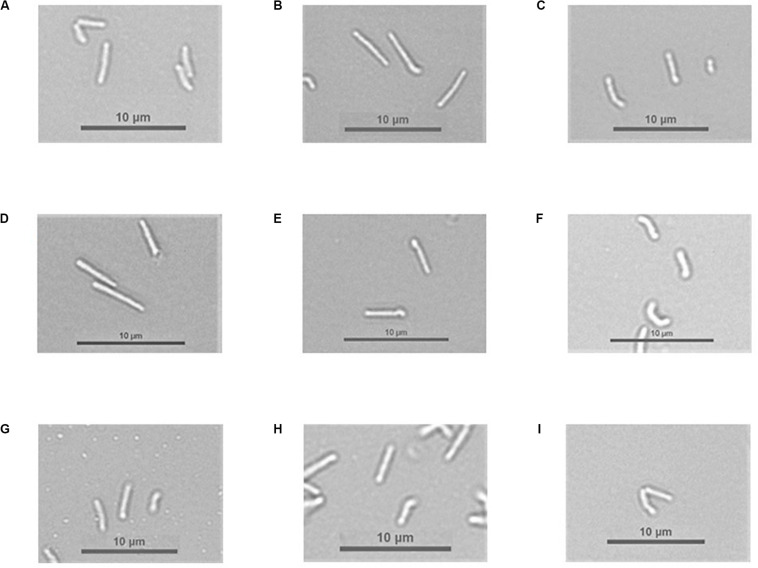
Bacterial cells under a light microscope. Pictures from **(A)** to **(F)** present *M. smegmatis* strains grown in 7H9 broth supplemented with cobalt chloride, OADC, and Tween 80. Pictures from **(G)** to **(I)** present *M. smegmatis* strains grown in 7H9 broth supplemented with cobalt chloride, OADC, Tween 80, and vitamin B12. **(A)** *M. smegmatis* mc^2^ 155 **(B)** Δ*4305* **(C)** Δ*cobIJ* **(D)** Δ*4305*:*attB* + *N’ter4305* **(E)** Δ*4305*:*attB* + *4305* **(F)** Δ*cobIJ*:*attB* + *cobIJ* **(G)** *M. smegmatis* mc^2^ 155 **(H)** Δ*4305* **(I)** Δ*cobIJ*.

First, we assessed mutant cell length compared to the wild strain by microscopic analysis (Figures 4, 5) and by the analysis of changes in FSC value on flow cytometer (Supplementary Figure S4). We observed statistically significant differences in length for Δ4305 and Δ*rnhA*Δ4305:*attB* + N’ter4305 strain when compared with the wild type during growth on 7H9 medium supplemented with cobalt (ANOVA *p* < 0.001, Dunnett’s *post hoc p* < 0.001 and *p* = 0,049, respectively). Mutants were approximately 1.3 times longer than *M. smegmatis* mc^2^ 155. The phenotype of the Δ4305 strain reversed upon supplementation of medium with vitamin B12. We observed no analogous change in the phenotype of the Δ*cobIJ* mutant. We confirmed the results obtained by light microscopy by the analysis of the FSC parameter obtained using the flow cytometer. Δ4305 and Δ*rnhA*Δ4305:*attB* + N’ter4305 showed a statistically significant increase in the FSC parameter (ANOVA *p* < 0.001, Dunnett’s *post hoc p* < 0.001).

**FIGURE 6:**
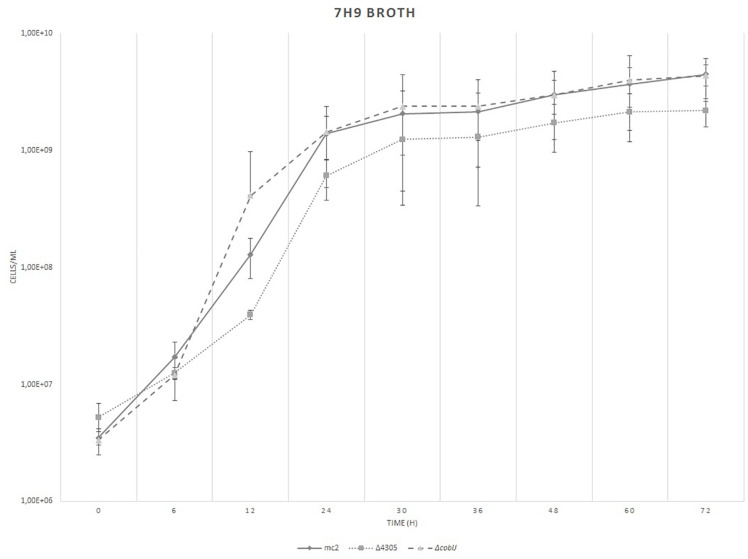
The growth of bacteria on rich broth, 7H9 supplemented with OADC, Tween 80, and cobalt chloride. We did not find statistically significant differences in growth rate between the strains. The growth curve was constructed by measuring the cell concentration of *M. smegmatis* strains on flow cytometer at designated time points for 3 days, with initial OD_600_ = 0.05. The data are representative of three independent experiments. Statistical analysis was performed by comparing cell density at different time points by one-way ANOVA. The cut-off level of statistical significance was *p* < 0.05.

Next, we assessed the growth rate of *M. smegmatis* mc^2^, Δ4305, and Δ*cobIJ* on rich broth, 7H9 supplemented with OADC, Tween 80, and cobalt. We did not detect any statistically significant differences between the number of CFU in mutant strains at different time-points by both flow cytometry (Figure 6) and by solid media plating (Supplementary Figure S5).

### CobC Domain of MSMEG_4305 Is Involved in Vitamin B12 Synthesis *in vivo*

**FIGURE 7:**
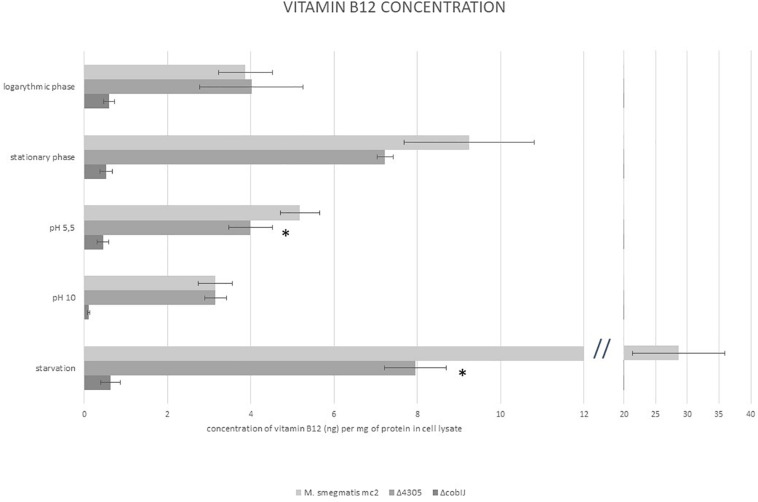
The concentration of vitamin B12 in whole-cell lysates of *M. smegmatis* determined by ELISA immunoassay. The data are representative of three independent experiments. Statistical analysis was performed by comparison of vitamin B12 level in cell lysates of *M. smegmatis* of analyzed strains with *M. smegmatis* mc2 by one-way ANOVA and Dunnett’s *post hoc*. The cut-off level of statistical significance was *p* < 0.05. Statistical significance is marked for Δ4305.

We detected vitamin B12 in cell lysates of *M. smegmatis* grown in various culture conditions (Figure 7). We used samples of cultures of Δ*cobIJ* strain as negative control for the test. We analyzed samples of cultures from logarithmic and stationary phases of growth, samples grown in acidified and alkaline medium, and samples obtained from starved cultures. Vitamin B12 was detected in all samples of *M. smegmatis* mc^2^ and Δ4305. All samples of Δ*cobIJ* contained less than 1 ng f vitamin B12 per mg of protein (range 0.076–0.958). Hence, the sensitivity of the method was determined at one ng per mg of protein lysate. For wild type *M. smegmatis*, logarithmic phase cultures, on average contained 3.869 ± 0.461 SE ng of vitamin B12 per mg of protein. The concentration of vitamin B12 increased significantly in starved cultures (ANOVA *p* < 0.001, Dunnett’s *post hoc p* < 0.001), when compared to logarithmic phase cultures, approximately by eightfold. Neither acidic (Dunnett’s *post hoc p* = 0.984) nor alkali growth medium (Dunnett’s *post hoc p* = 0.998) affected the concentration of vitamin B12 inside *M. smegmatis* mc^2^. The deletion of MSMEG_4305 significantly affected the concentration of vitamin B12 in acidic (ANOVA *p* < 0.001, Dunnett’s *post hoc p* = 0.049) and starved cultures (ANOVA *p* < 0.001, Dunnett’s *post hoc p* = 0.005) when compared with wild type.

Sulphonamides target folate synthesis *de novo*. Bacteria with impaired synthesis of vitamin B12 are hyper-susceptible to sulphonamides, due to the formation of so-called methylfolate trap. The methylfolate trap is a biochemical block, which results from the inability to complete folate recycling. One of the enzymes of the folate cycle is a vitamin B12 dependent methionine synthase (Guzzo et al., 2016). Methionine synthase catalyzes the transfer of a methyl group from methyltetrahydrofolate to homocysteine to produce methionine and tetrahydrofolate. Methyl-cobalamin acts as both an acceptor and donor of the methyl group.

**FIGURE 8:**
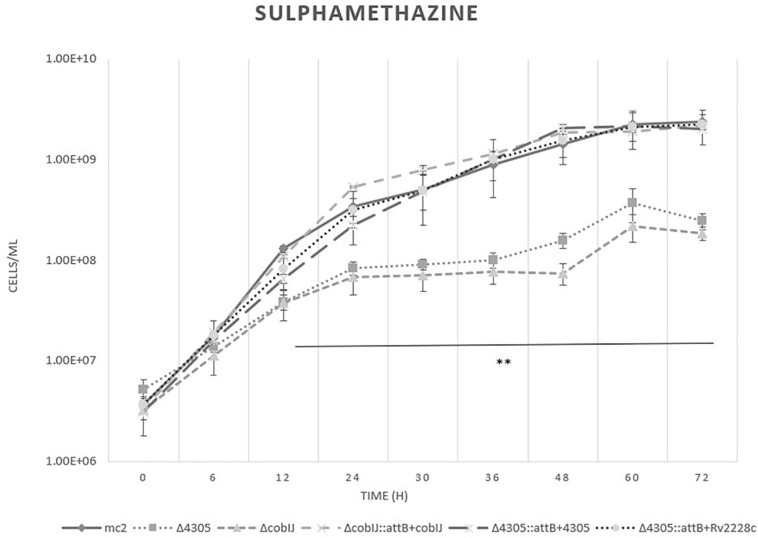
The growth curves of *M. smegmatis* strains grown in 7H9 broth supplemented with cobalt chloride, sulfamethazine, OADC and Tween 80. The growth curve was constructed by measuring the cell concentration of *M. smegmatis* strains on flow cytometer at designated time points for 3 days, with initial OD_600_ = 0.05. The data are representative of three independent experiments. Statistical analysis was performed by comparing cell density at different time points by one-way ANOVA and Dunnett’s *post hoc*. The cut-off level of statistical significance was *p* < 0.05. Two asteriscs mark two *M. smegmatis* strains statistically different from wild type.

To further investigate the role of MSMEG_4305 in vitamin B12 biosynthesis, we screened the growth of MSMEG_4305 on sulfamethazine (Figure 8). The growth of the Δ4305 strain was restricted, as well as the growth of Δ*cobIJ*. These results suggest the formation of a methylfolate trap within the cells of the mutant strains. Complementation of mutant strains with intact copies of the genes at the *attB* site restored their growth to wild type. Drug susceptible phenotype of MSMEG_4305 deficient mutant was also complemented with Rv2228c. The results obtained by flow cytometry were confirmed by plating batches of cultures on solid media (Supplementary Figure S6).

**FIGURE 9:**
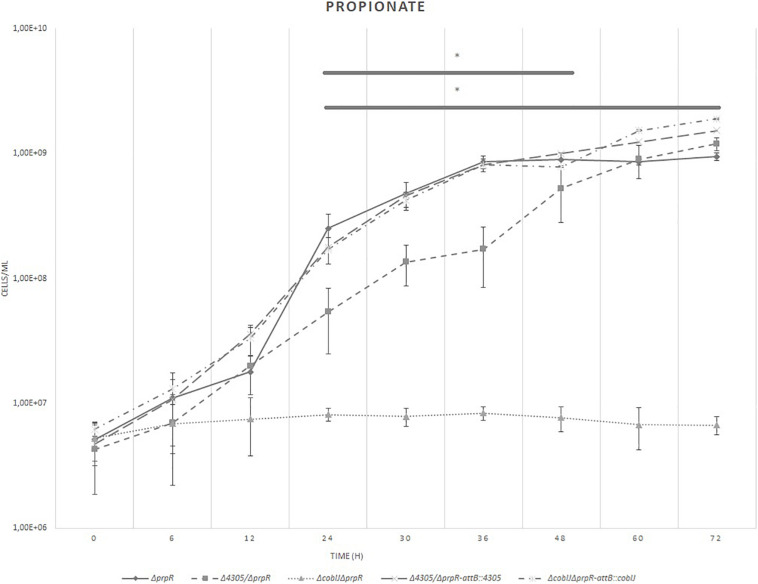
The growth curves of *M. smegmatis* strains grown in minimal broth supplemented with cobalt chloride, propionate, and tyloxapol. The growth curve was constructed by measuring the cell concentration of *M. smegmatis* strains on flow cytometer at designated time points for 3 days, with initial OD_600_ = 0.05. The data are representative of three independent experiments. Statistical analysis was performed by comparing cell density at different time points by one-way ANOVA and Dunnett’s *post hoc*. The cut-off level of statistical significance was *p* < 0.05.

To further investigate the ability of Δ4305 to synthesize vitamin B12 we tested the ability of the mutant to grow on propionate as a sole carbon source in Δ*prpR* background (Figure 9 and Supplementary Figures S1, S7). It was shown previously, that mycobacteria utilize two distinct pathways to metabolize propionate- the methylmalonyl pathway, involving vitamin B12 dependent methylmalonyl CoA mutase and isocitrate cycle, metabolized by PrpC and PrpD, controlled by PrpR (Masiewicz et al., 2012).

As a control, the strains were grown on minimal medium supplemented with glucose, cobalt chloride, and tyloxapol. We did not observe significant differences between the strains (Supplementary Figures S8, S9). When grown on propionate, we observed that the growth of Δ4305 was significantly delayed compared with the wild type starting at 24 h of culture (ANOVA *p* = 0.001, Dunnett’s *post hoc p* = 0.004). Its growth was not entirely restricted as it was for Δ*cobIJ* mutant (ANOVA *p* = 0.001, Dunnett’s *post hoc p* < 0.001). Complementation of mutant strains with intact copies of the genes at the *attB* site reversed the phenotype of the mutant strains to the wild type.

### Disassociation Between CobC Domain on RNase H Domain of MSMEG_4305 *in vivo*

Double deletion of RNases H type I, simultaneous knock-out Δ4305 and Δ*rnhA*, is not tolerated in *M. smegmatis*, probably due to the accumulation of RNA/DNA hybrids, which cause replication fork stalling and DNA breaks. It was not possible to exchange complementation episome in Δ*rnhA*Δ4305:*attB* + 4305 for an empty vector (Minias et al., 2015a). In this study we obtained mutants deficient in Δ*rnhA* Δ4305:*attB* + Rv2228c (Supplementary Figure S3).

**FIGURE 10:**
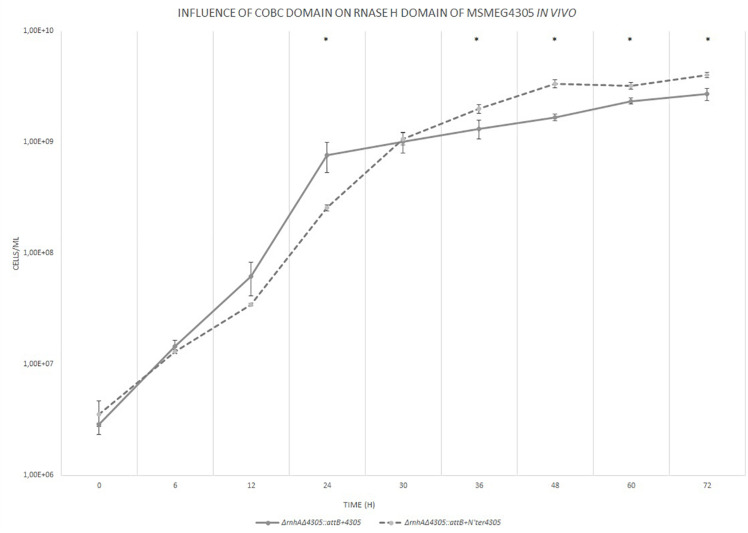
The growth curves of *M. smegmatis* strains grown in 7H9 broth supplemented with OADC, cobalt chloride and Tween 80. The growth curve was constructed by measuring the cell concentration of *M. smegmatis* strains on flow cytometer at designated time points for 3 days, with initial OD_600_ = 0.05. The data are representative of three independent experiments. Statistical analysis was performed by comparing cell density at different time points by Student’s *t*-test. The cut-off level of statistical significance was *p* < 0.05.

We tested whether the CobC domain influences RNase HI domain *in vivo* by constructing a mutant strain Δ*rnhA*Δ4305: *attB* + N’ter4305 expressing truncated MSMEG_4305 in Δ*rnhA* background and by analyzing its phenotype (Figure 10). The mutant Δ*rnhA*Δ4305:*attB* + N’ter4305 showed a slight, yet statistically significant, delay in growth at 24 h of culture (*t* = 3.776, df = 4, *p* = 0.019). The delay at 24 h of growth was also observable when we used a solid media plating method to assess the number of CFU (*t* = 7.859, df = 4, *p* = 0.001) (Supplementary Figure S10). The mutant eventually reached a higher density at 36 h of growth. The increase in the biomass was relatively minor, 1.5 fold increase in the CFU, and it was observable only on the flow cytometer.

**FIGURE 11:**
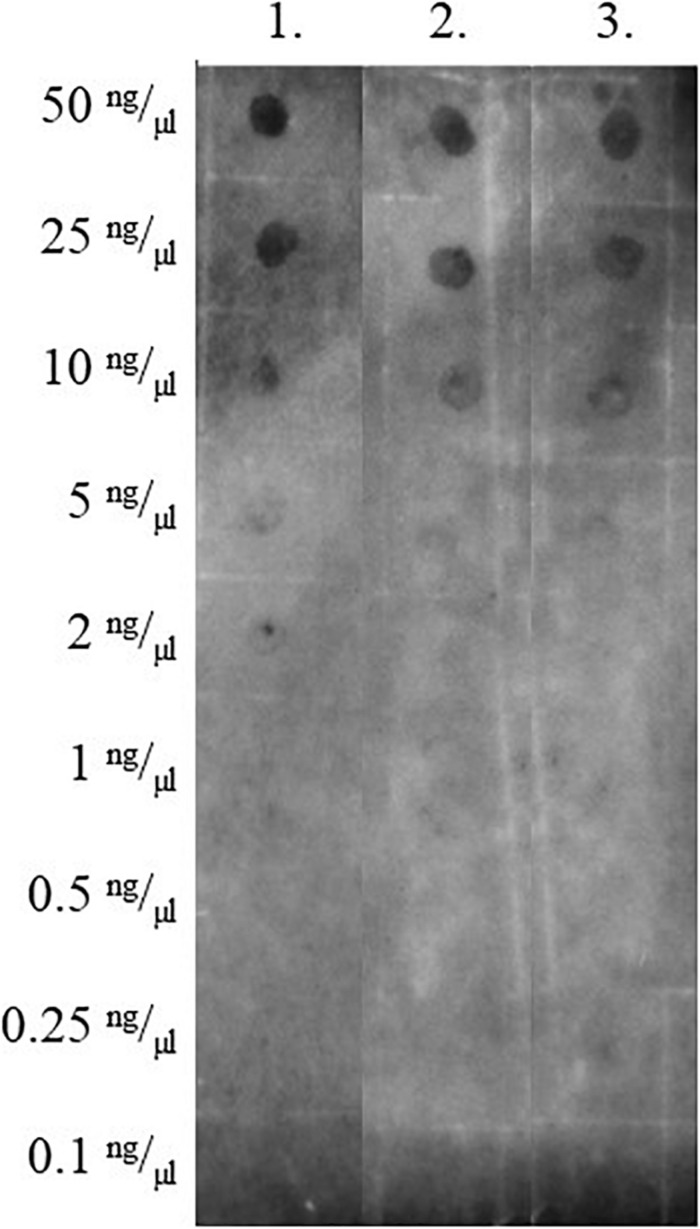
Comparison of the level of RNA/DNA hybrids in nucleic acids isolated from cells from strains of *M. smegmatis*. Nucleic acids were placed on a nylon membrane and detected with antibodies specific to the RNA/DNA complex. 1. *M. smegmatis* mc^2^ 155, 2. Δ*rnhA*Δ4305:*attB* + 4305 3. Δ*rnhA*Δ4305:*attB* + N’ter4305.

**FIGURE 12:**
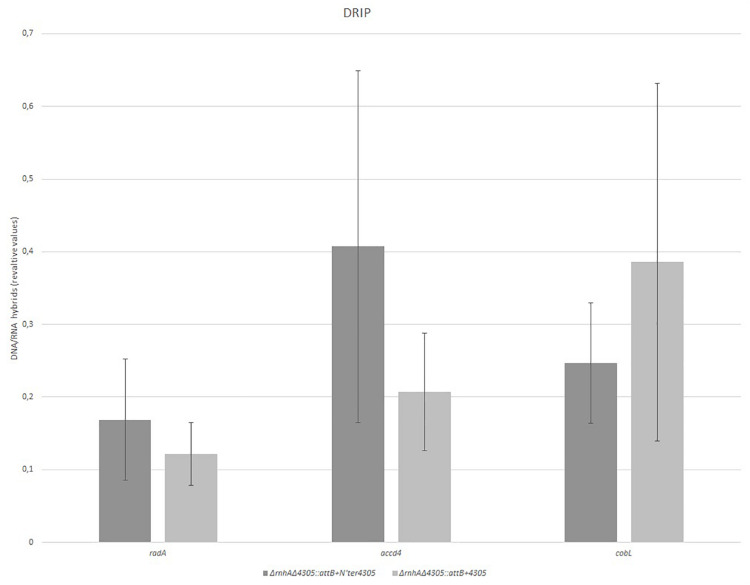
Comparison of the level of RNA/DNA hybrids in Δ*rnhA*Δ4305:*attB* + 4305 and Δ*rnhA*Δ4305:*attB* + N’ter4305. RNA/DNA hybrids were immunoprecipitated with an S9.6 antibody. qPCR allowed the estimation of RNA/DNA hybrids in precipitate in reference to the corresponding input. The data are representative of three experiments. Statistical analysis was performed by Student’s *t*-test. The cut-off level of statistical significance was *p* < 0.05. We found no statistically significant difference in the level of RNA/DNA hybrids in both strains (*t* = 0.711, df = 4, *p* = 0.517 for *radA*; *t* = 1.105, df = 4, *p* = 0.331 for *accD4*, and *t* = 0.757, df = 4, *p* = 0.491 for *cobL*).

Next, we wanted to see if there was a change in the level of RNA/DNA hybrids in the mutant Δ*rnhA*Δ4305:*attB* + N’ter4305. We used a modified Western dot blot technique to detect RNA/DNA hybrids in whole-cell nucleic acids (Figure 11). The level of RNA/DNA hybrid level was similar in Δ*rnhA*Δ4305:*attB* + N’ter4305 and control strains Δ*rnhA*Δ4305:*attB* + 4305 and wild type *M. smegmatis* mc^2^. We confirmed that there was no statistically significant increase in the level of RNA/DNA hybrids in Δ*rnhA*Δ4305:*attB* + N’ter4305 by immunoprecipitation and subsequent qPCR (Figure 12).

Finally, we analyzed the competitive fitness of mutants. The results showed that the mutant Δ*rnhA*Δ4305:*attB* + N’ter4305 had relative fitness to Δ*rnhA*Δ4305:*attB* + 4305 of 1.21 ± SE 0.219 at 24 h of mixed culture and 1.145 ± SE 0.113 at 72 h of mixed culture.

## Discussion

We analyzed the phenotype related to vitamin B12 biosynthesis of *M. smegmatis* deficient in *Actinomycetales* specific protein, MSMEG_4305, and we investigated whether there is a functional association between domains of the MSMEG_4305 protein *in vivo*. The analyzes were carried out by determining the number of CFU by plating onto solid-plate plates and using a flow cytometer. The results of the methods were comparable. The minor differences between the readings obtained by flow cytometry and by solid cell plating result from the characteristic features of both methods. For example, cell plating on solid media does not identify the subpopulations of cells that are in the non-culturable state. Further, cell plating on solid media requires serial dilution of cell suspension, with a significant challenge in sampling and counting errors (Ben-David and Davidson, 2014). In turn, results obtained by flow cytometry, turbidimetry, and optical density can be distorted, for example, due to the presence of dead and damaged cells in the suspension. Therefore, while a general trend in bacterial growth kinetics obtained by distinct methods should be similar, certain minor discrepancies are possible.

Δ4305 cells and Δ*rnhA*Δ4305:*attB* + N’ter4305 cells deficient in the C-terminal domain of MSMEG_4305 are elongated compared to the wild strain. The mutant phenotype is reversed to the wild strain after supplementation of medium with vitamin B12. Since morphological changes reversed upon supplementation of the medium with vitamin B12, the phenotypic changes observed in the mutants are associated with the deletion of the CobC domain, not the RNase domain.

Vitamin B12 concentration diminished in cell lysates obtained from Δ4305 *M. smegmatis* cultures in acidic medium and medium with limited carbon source when compared with wild type *M. smegmatis* mc^2^. The Δ4305 showed a delay in growth on medium supplemented with sulphonamide and showed growth impairment during growth on propionate in Δ*prpR* background. These results confirm that the CobC domain of MSMEG_4305 is involved in vitamin B12 synthesis *in vivo* under necessary conditions. Several species of bacteria within *Actinomycetales* maintain an additional CobC encoding gene (*M. tuberculosis* Rv2231c, *M. avium* AIV24907.1, and *M. abscessus* MAB_1902). We suspect that as in other species of mycobacteria, the genome of *M. smegmatis* probably encodes another protein that can confer the function of the CobC domain of MSMEG_4305. However, this mystery protein is not sufficient to provide enough vitamin B12 substrates for optimal vitamin B12 synthesis in certain conditions. Since the major limitation in vitamin B12 production in Δ4305 occurred in medium with limited carbon source and in acidified medium, we suspect that the alternative CobC optimally functions during active growth and in the alkali medium. The bioinformatic screen of Mycobrowser Database identifies three potential alkaline phosphatases in the genome of *M. smegmatis* mc^2^- MSMEG_5508, MSMEG_1012, MSMEG_2292. The database also identifies one additional acid phosphatase- MSMEG_6820 (annotated as SurE).

Introduction of Rv2228c into strain deficient in MSMEG_4305 allowed to rescue phenotype related to vitamin B12 production and allowed for deletion of both RNases H type I encoded by *M. smegmatis* genome. We conclude that Rv2228c complements the function of of MSMEG_4305 and it is homologous to this protein.

The deletion of the CobC domain of MSMEG_4305 in Δ*rnhA* background resulted in very slight growth delay observable by flow cytometry and by CFU plating. The delay was only observable at 24 h of growth. There were no differences in growth rate when Δ*rnhA*Δ4305:*attB* + N’ter4305 and Δ*rnhA*Δ4305:*attB* + 4305 strains grew against each other in the competition assay. There was no statistically significant increase in the level of RNA/DNA hybrids in Δ*rnhA*Δ4305:*attB* + N’ter4305. The delay might have been caused by the necessity to process the RNA/DNA hybrids without the RNase HI of MSMEG_4305, which is more evident during the active phase of growth. Alternatively, the minor decrease in the growth rate of Δ*rnhA*Δ4305:*attB* + N’ter4305 might be a result of the disorganization of physical inter-domain interactions generated thru truncation of CobC domain. These interactions involve the stabilization of the domains by their natural neighbors and have previously been described, for example, for tandem repeat proteins (MacDonald and Pozharski, 2001). The protein stabilization often occurs primarily by hydrophobic residues in the inter-domain interface, thereby stabilizing otherwise unstable domains (Bhaskara and Srinivasan, 2011).

Genome evolution seems to be largely a stochastic process, which is modulated by natural selection (Koonin et al., 2002). The promiscuity of certain protein domain combinations across the tree of life is mainly a result of inheritance, either driven by selective pressure or genetic drift. Further, there is evidence of the convergent evolution of domain combinations driven by selective pressure (Apic et al., 2001; Gough, 2005; Forslund et al., 2008; Basu et al., 2009). While it is generally accepted that multidomain combinations can lead to the generation of new functions within cells (Bashton and Chothia, 2007), it remains to be established how many of the combined domains interact and influence each other. New functions of multidomain proteins can be acquired through sequence divergence, for example, thru point mutations (Nahum and Riley, 2001). Changes in sequence can alter ligand specificity and create a platform for different kinds of biochemical reactions (Nahum and Riley, 2001). Another way of creating new functions is domain combination, which makes multidomain proteins more specific or more complex in their functions than their monomer counterparts (Bashton and Chothia, 2007). Domain arrangements, even the domain order in multidomain proteins, determine the 3D structure of the proteins and hence might affect their function (Bashton and Chothia, 2002).

The evolutionary patterns among MSMEG_4305 homologs show that both domains are under purifying selective pressure, which suggests that the presence of both functional domains is adaptive for the cell. However, the level of nucleotide substitutions and the overall mean distance among homologs of MSMEG_4305 across different species of bacteria both suggest that RNase H domains are less variable and are under stronger influence of purifying selection. These observations link with the essential function of RNase H, which is involved in fundamental metabolic processes in the cells. In turn, the presence of vitamin B12, while adaptive, it is dispensable (Minias et al., 2018). The linker region between the domains of homologs of MSMEG_4305 across the *Actinomycetales* species is highly variable. Likely it does not play a significant role in maintaining the functionality of the entire protein.

Estimations show that 65% of proteins in eukaryotes and 40% of proteins of Prokaryotes are multidomain (Ekman et al., 2005). The reasons behind the evolution of the multidomain organization of proteins are still under investigation. Many domains in eukaryotic multidomain proteins are found as independent proteins in prokaryotes. It was suggested that a more frequent occurrence of multidomain organization of eukaryotic proteins provides an advantage of coordinate expression of enzymes, which is provided by the operon structure in bacteria. Hence, distinct strategies used by eukaryotes and prokaryotes to express coordinately regulated genes are dictated by the differences in the translational mechanisms (Zhou et al., 2019). RNase H domain forms a larger functional complex with the polymerase domain in reverse transcriptases, for example, in human immunodeficiency virus (HIV) (Sarafianos et al., 2009). Both domains of reverse transcriptases are involved in a single process of transferring genetic information encoded in the RNA into the DNA. For the MSMEG_4305, while it seems that both domains are involved in distinct processes, they are both involved in nucleotide metabolism. The CobC domain is involved through the folate cycle. The RNase H domain is involved through RNA degradation. Both domains are, therefore, particularly required for the active metabolic state of the cell. The co-existence of both domains in MSMEG_4305 may provide an advantage for the cell in terms of the coordinated expression of the genes required at a particular stage of the life cycle. Alternatively, the co-existence of both domains in MSMEG_4305 is not detrimental to cells, and therefore such combination was fixed in descendants of *Actinomycetales* through genetic drift.

In summary, we confirm that the CobC domain of MSMEG_4305 is involved in vitamin B12 biosynthesis. The truncation of CobC domain hardly affects the survival of *M. smegmatis* in Δ*rnhA* background. We conclude that MSMEG_4305 is a multidomain protein that most probably was fixed in the phylogenetic tree of life due to genetic drift.

## Data Availability Statement

The raw data generated for this study are available upon request to the corresponding author.

## Author Contributions

AM and JD designed the study. BC, AM, AŻ, and KS carried out the experiments. BC, AM, and JD wrote the manuscript. JZ-C performed critical revision. AM and JD supervised the project. All authors contributed to the article and approved the submitted version.

## Conflict of Interest

The authors declare that the research was conducted in the absence of any commercial or financial relationships that could be construed as a potential conflict of interest.
