# Supplementary figures and images for: Functional Disassociation Between the Protein Domains of MSMEG_4305 of Mycolicibacterium smegmatis (Mycobacterium smegmatis) in vivo
Source: Front Microbiol. 2020 Aug 19;11:2008. doi: 10.3389/fmicb.2020.02008 (PMC7466739; doi:10.3389/fmicb.2020.02008)

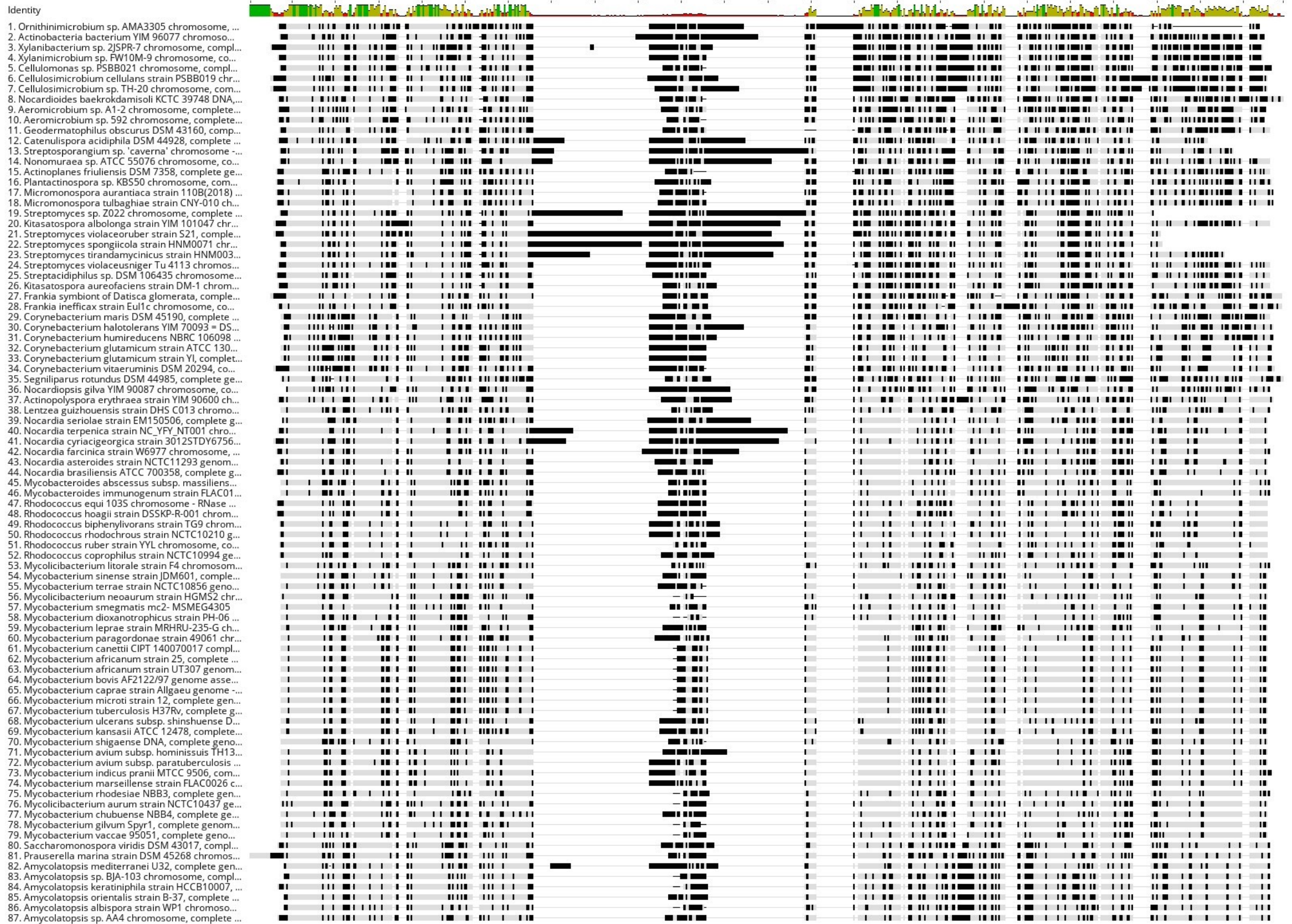

Supplement: Supplementary file 3 [file Data_Sheet_1.PDF]
