# Supplementary material for: Functional Disassociation Between the Protein Domains of MSMEG_4305 of Mycolicibacterium smegmatis (Mycobacterium smegmatis) in vivo
Source: Front Microbiol. 2020 Aug 19;11:2008. doi: 10.3389/fmicb.2020.02008 (PMC7466739; doi:10.3389/fmicb.2020.02008)
Supplement: Supplementary file 4 [file Data_Sheet_2.PDF]

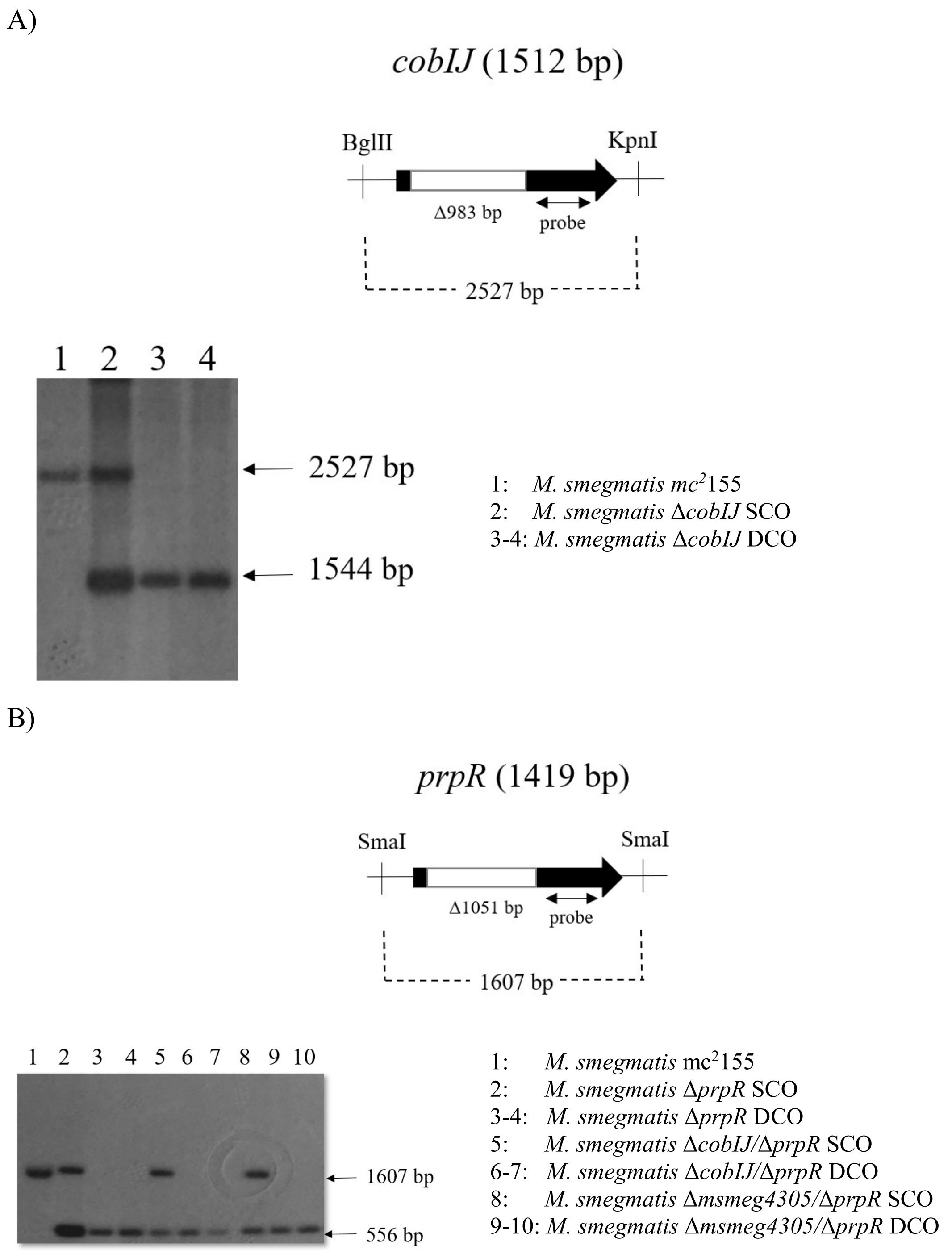

Fig S2. Southern blots confirming knock-out of genes A) *cobIJ* (MSMEG_3873) and B) *prpR* (MSMEG_6643) in *M. smegmatis*.
