# Supplementary material for: Functional Disassociation Between the Protein Domains of MSMEG_4305 of Mycolicibacterium smegmatis (Mycobacterium smegmatis) in vivo
Source: Front Microbiol. 2020 Aug 19;11:2008. doi: 10.3389/fmicb.2020.02008 (PMC7466739; doi:10.3389/fmicb.2020.02008)
Supplement: Supplementary file 5 [file Data_Sheet_3.PDF]

A) amplification of *Rv2228c* (1095 bp)

1. - 4.: clones *M. smegmatis* Δ*msmeg4305::attB+Rv2228c*
5.: pMV306Km+Rv2228c
6.: *M. smegmatis* mc^2

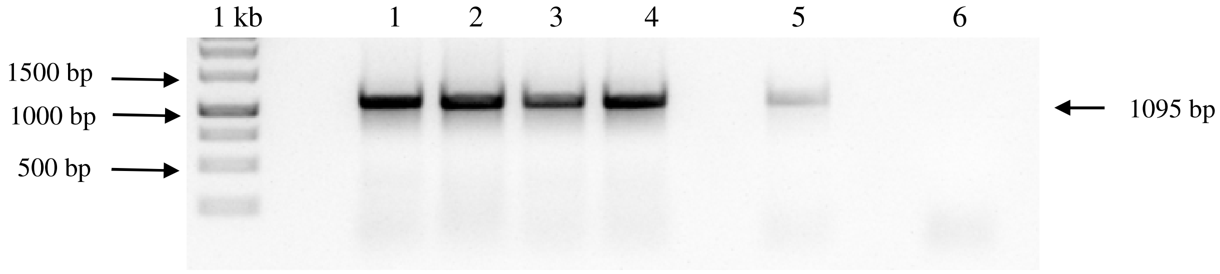

B) amplification of *msmeg4305* (wild type 1098 bp, deletion strain 379 bp)

1. - 2.: clones *M. smegmatis* Δ*rnhA*/Δ*msmeg4305::attB+Rv2228c*
3.: *M. smegmatis* mc^2
4.: *M. smegmatis* Δ*msmeg4305*

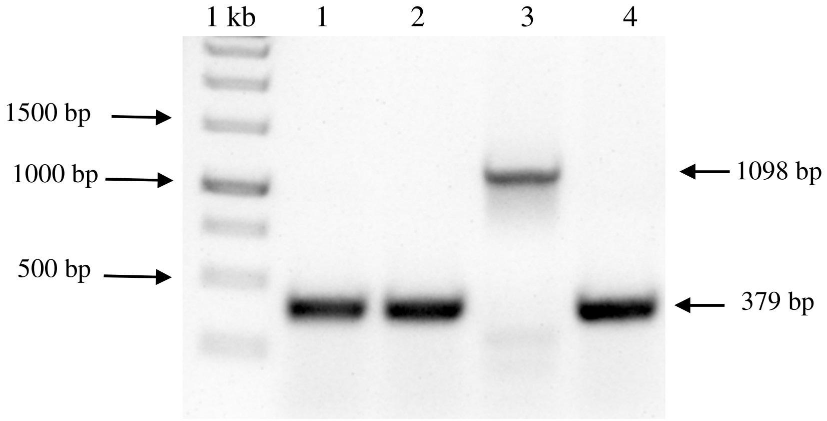

C) amplification of *Rv2228c* (1095 bp)

1. - 2.: *M. smegmatis* Δ*rnhA*/Δ*msmeg4305::+Rv2228c*
3.: *M. smegmatis* mc^2
4.: *M. smegmatis* Δ*rnhA*/Δ*msmeg4305::+4305*

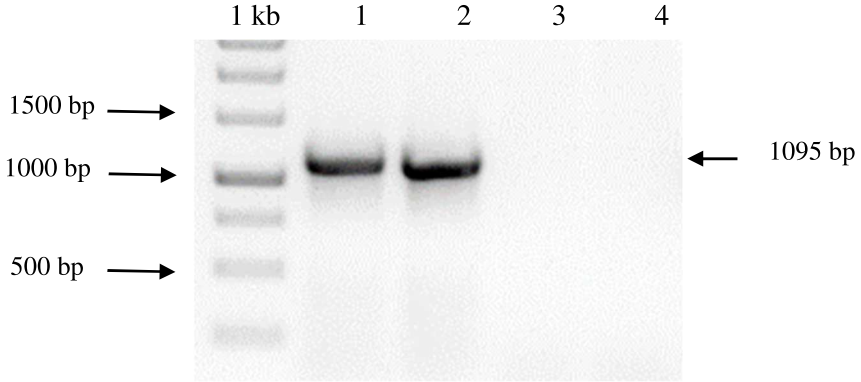

Fig. S3. PCR amplification of gene products confirming introduction of Rv2228c complementation plasmid into *M. smegmatis*. Fig. B) and Fig. C).depict the same clones 1 and 2 of

ΔrnhA/Δmsmeg4305::+Rv2228c. Amplification of MSMEG_4305 allowed to control potential reintroduction of the wild type MSMEG_4305 back to the native site at the time of complementation with Rv2228c. PCR products of *ΔrnhA/Δmsmeg4305::+Rv2228c* amplified with primers targeting MSMEG_4305 show that the native site contains mutant version of MSMEG_4305.
