# Supplementary material for: Functional Disassociation Between the Protein Domains of MSMEG_4305 of Mycolicibacterium smegmatis (Mycobacterium smegmatis) in vivo
Source: Front Microbiol. 2020 Aug 19;11:2008. doi: 10.3389/fmicb.2020.02008 (PMC7466739; doi:10.3389/fmicb.2020.02008)
Supplement: Supplementary file 6 [file Data_Sheet_4.pdf]

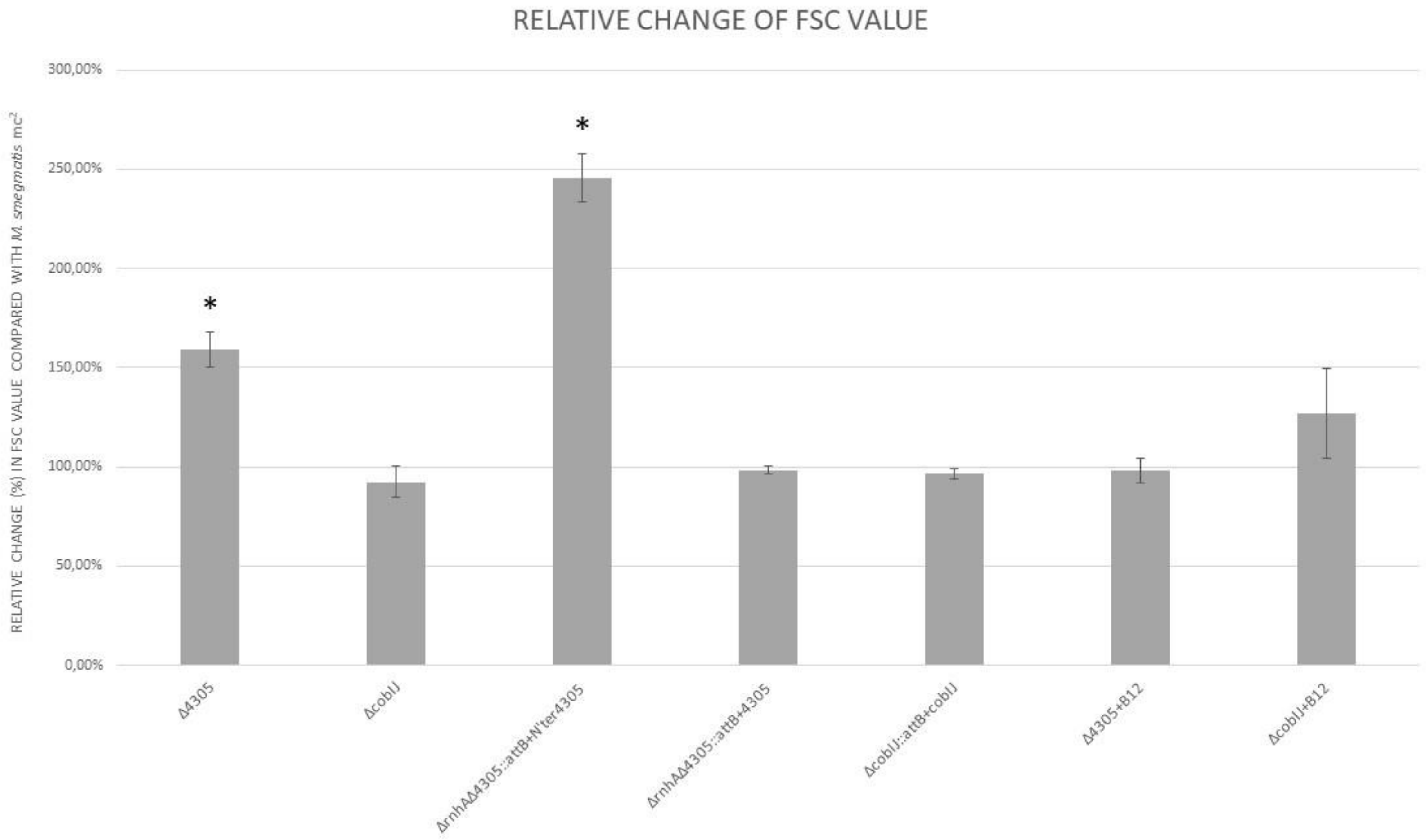

Fig. S4. Relative change of the FSC value of *M. smegmatis* strains grown in 7H9 broth supplemented with cobalt chloride, OADC, and Tween 80. The data are representative of three independent experiments. Statistical analysis was performed by comparison of the FSC value of analyzed strains with *M. smegmatis* mc2 by one-way ANOVA and Dunnett's post hoc. The cut-off level of statistical significance was $p < 0.05$.
