# Supplementary material for: Functional Disassociation Between the Protein Domains of MSMEG_4305 of Mycolicibacterium smegmatis (Mycobacterium smegmatis) in vivo
Source: Front Microbiol. 2020 Aug 19;11:2008. doi: 10.3389/fmicb.2020.02008 (PMC7466739; doi:10.3389/fmicb.2020.02008)
Supplement: Supplementary file 7 [file Data_Sheet_5.pdf]

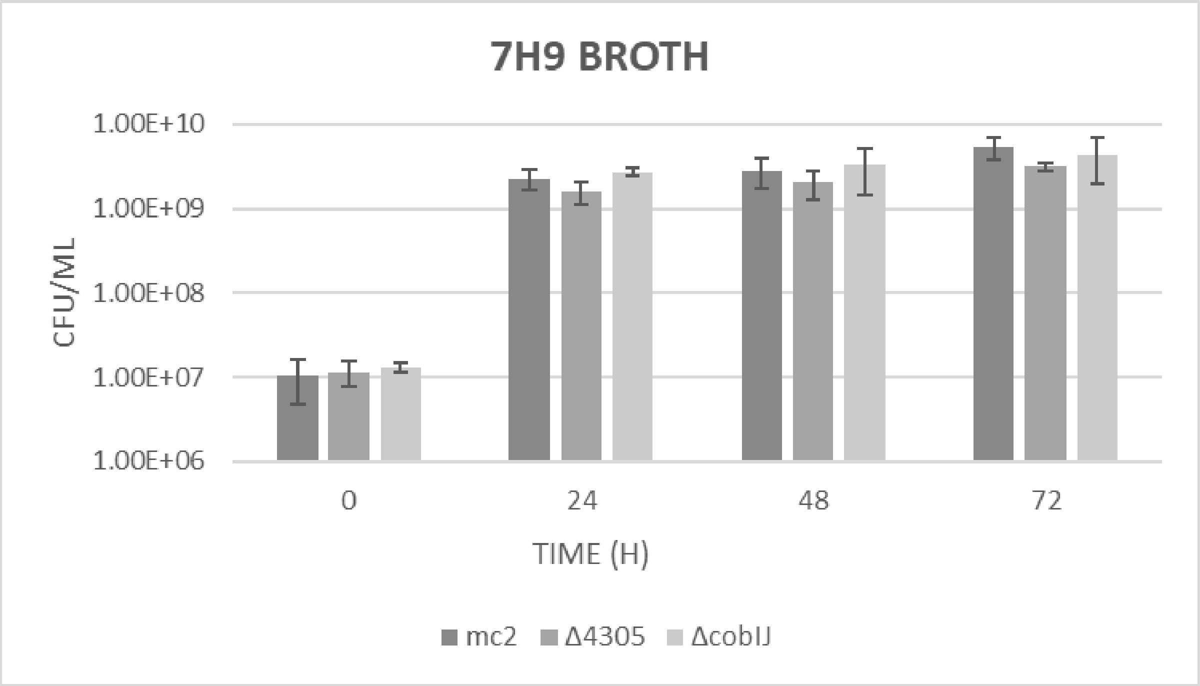

Fig. S5. Evaluation of the number of colony-forming units (CFU) by classical plating method during growth in rich medium 7H9 broth supplemented with cobalt chloride, OADC and Tween 80. The data are representative of three independent replicates. Statistical analysis was performed by comparing cell density at different time points by one-way ANOVA. We did not find statistically significant differences between the strains at different time points.
