# Supplementary material for: Functional Disassociation Between the Protein Domains of MSMEG_4305 of Mycolicibacterium smegmatis (Mycobacterium smegmatis) in vivo
Source: Front Microbiol. 2020 Aug 19;11:2008. doi: 10.3389/fmicb.2020.02008 (PMC7466739; doi:10.3389/fmicb.2020.02008)
Supplement: Supplementary file 9 [file Data_Sheet_7.pdf]

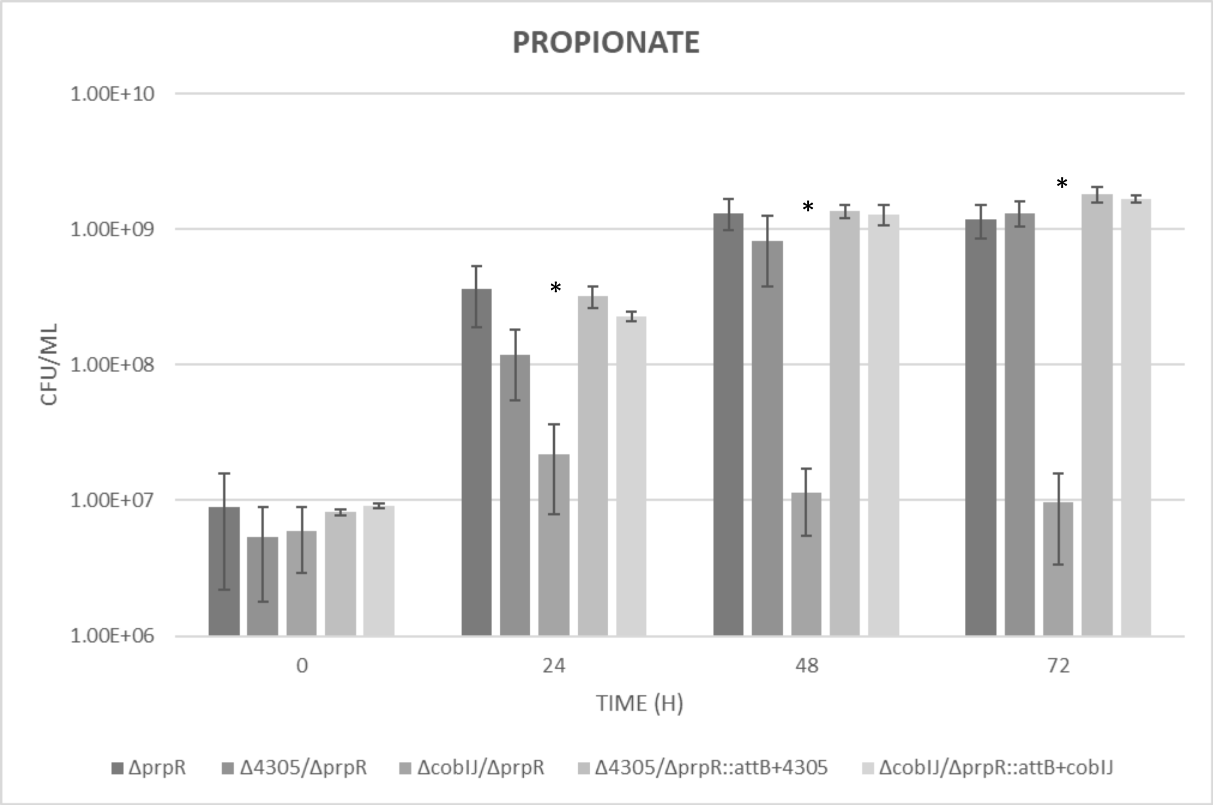

Fig. S7. Evaluation of the number of CFU by classical plating method during growth in minimal broth supplemented with propionate, cobalt chloride, and tyloxapol. The cultures were started at initial OD_{600}=0.05, and diluted batches were plated at designated time points for three days. The data are representative of three independent experiments. Statistical analysis was performed by comparing cell density at different time points by one-way ANOVA and Dunnett's post hoc. The cut-off level of statistical significance was $p<0.05$.
