# Supplementary material for: Functional Disassociation Between the Protein Domains of MSMEG_4305 of Mycolicibacterium smegmatis (Mycobacterium smegmatis) in vivo
Source: Front Microbiol. 2020 Aug 19;11:2008. doi: 10.3389/fmicb.2020.02008 (PMC7466739; doi:10.3389/fmicb.2020.02008)
Supplement: Supplementary file 10 [file Data_Sheet_8.pdf]

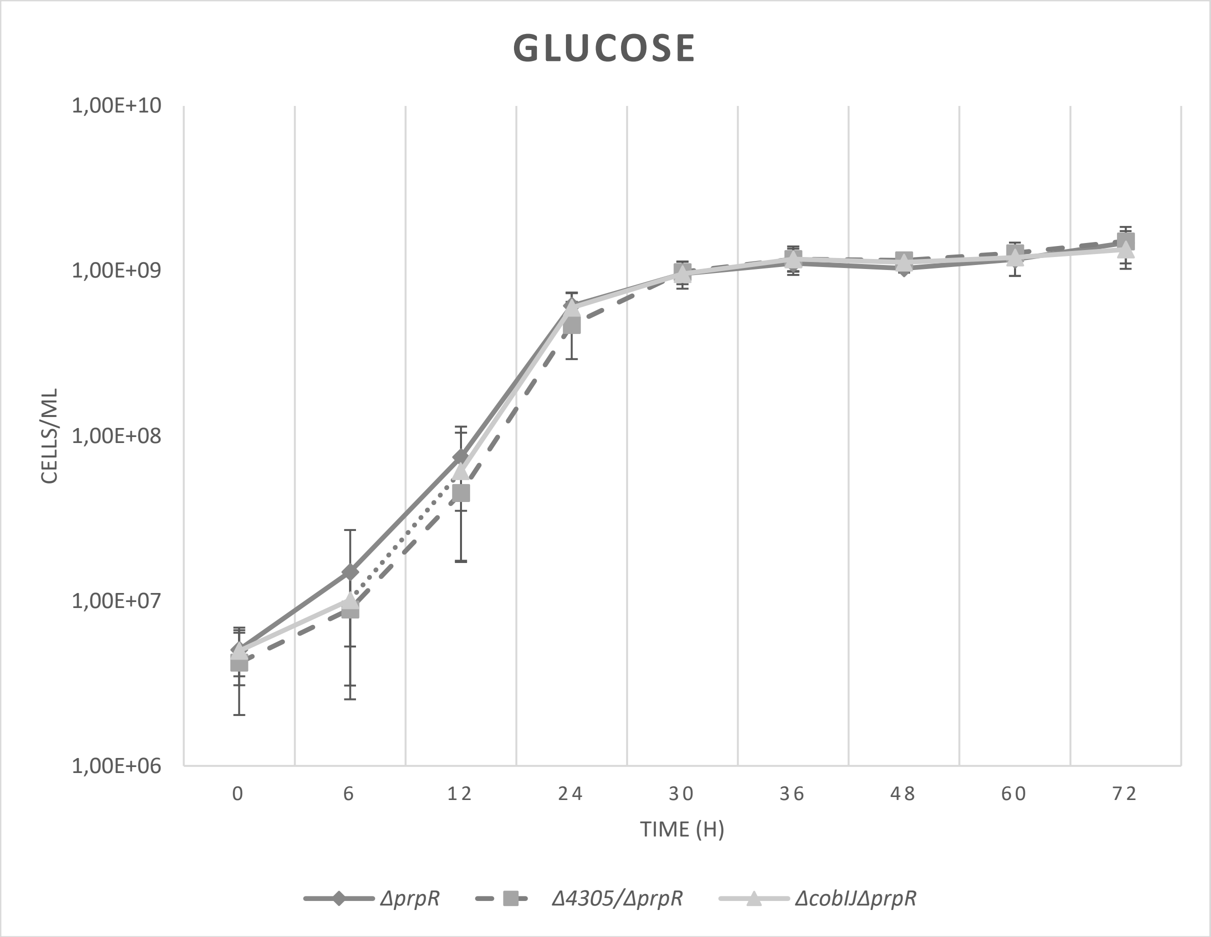

Fig. S8. The growth curve of *M. smegmatis* strains grown on minimal medium supplemented with glucose, cobalt chloride, and tyloxapol. The growth curve was constructed by measuring the cell concentration of *M. smegmatis* strains on flow cytometer at designated time points for three days, with initial OD_{600}=0.05. The data are representative of three independent replicates. Statistical analysis was performed by comparing cell density at different time points by one-way ANOVA. We did not find statistically significant differences between the strains at different time points.
