# Supplementary material for: Functional Disassociation Between the Protein Domains of MSMEG_4305 of Mycolicibacterium smegmatis (Mycobacterium smegmatis) in vivo
Source: Front Microbiol. 2020 Aug 19;11:2008. doi: 10.3389/fmicb.2020.02008 (PMC7466739; doi:10.3389/fmicb.2020.02008)
Supplement: Supplementary file 11 [file Data_Sheet_9.pdf]

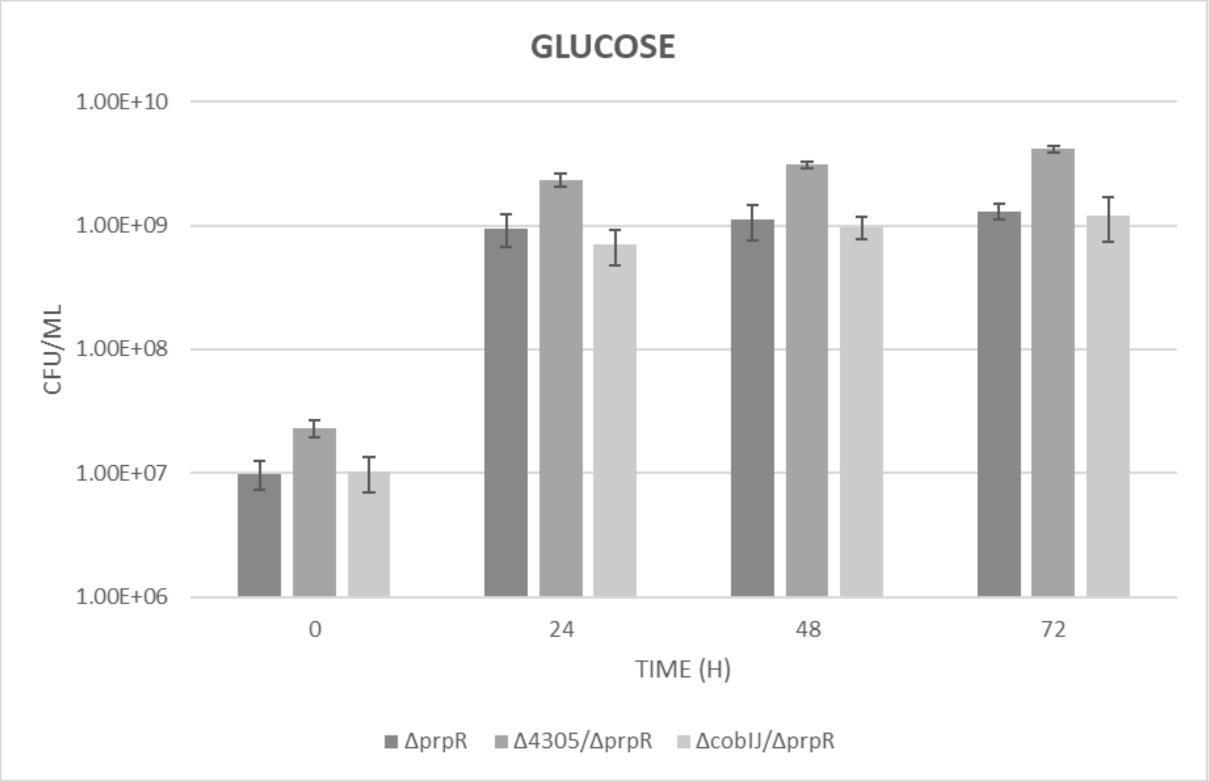

Fig. S9. Evaluation of the number of CFU by classical plating method during growth in minimal broth supplemented with glucose, cobalt chloride, and tyloxapol. The cultures were started at initial $OD_{600}=0.05$, and diluted batches were plated at designated time points for three days. The data are representative of three independent experiments. Statistical analysis was performed by comparing cell density at different time points by one-way ANOVA; the cut-off value of significance was $p<0.05$. We did not find any statistically significant differences in the growth rate between the strains.
