# Supplementary material for: Functional Disassociation Between the Protein Domains of MSMEG_4305 of Mycolicibacterium smegmatis (Mycobacterium smegmatis) in vivo
Source: Front Microbiol. 2020 Aug 19;11:2008. doi: 10.3389/fmicb.2020.02008 (PMC7466739; doi:10.3389/fmicb.2020.02008)
Supplement: Supplementary file 12 [file Data_Sheet_10.pdf]

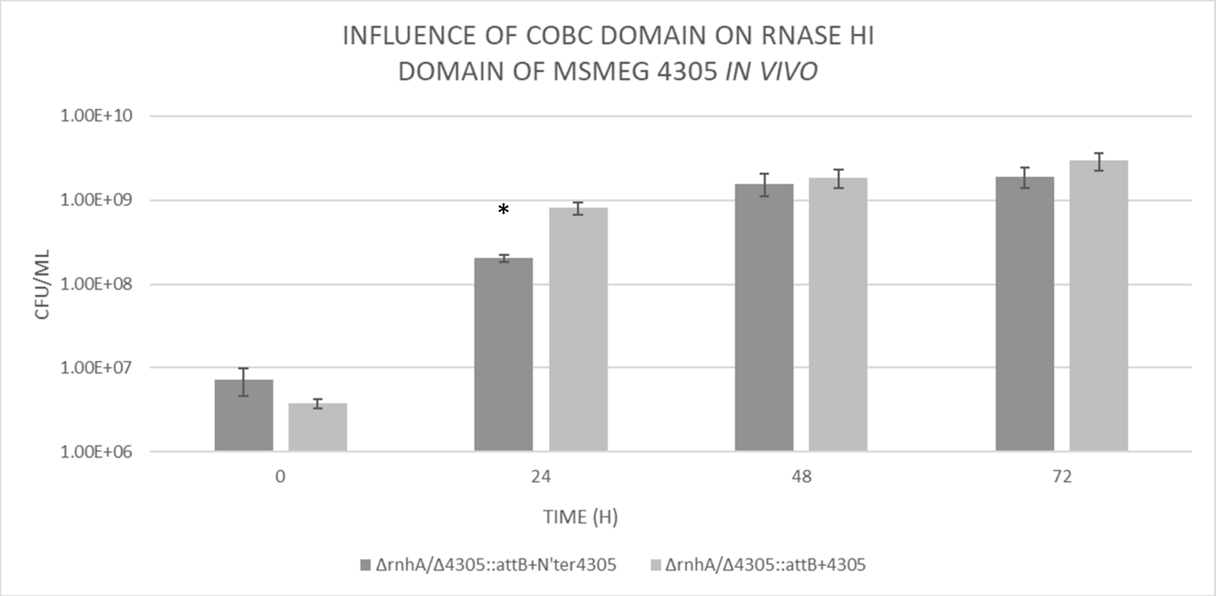

Fig. S10. Evaluation of the number of CFU by classical plating method during growth in 7H9 broth supplemented with OADC, cobalt chloride, and Tween 80. The cultures were started at initial OD_{600}=0.05, and diluted batches were plated at designated time points for three days. The data are representative of three independent experiments. Statistical analysis was performed by comparing cell density at different time points by Student’s t-test. The cut-off level of statistical significance was $p<0.05$..
